# Optimising Rehabilitation Strategies for Primary Central Nervous System Neoplasm-Related Neurological Deficits Using a Comprehensive Multimodality Approach Including Robotic Gloves Technology: A Comprehensive Case Report

**DOI:** 10.7759/cureus.56221

**Published:** 2024-03-15

**Authors:** Prajyot Ankar, Snehal Samal, Swarna Singh

**Affiliations:** 1 Neurophysiotherapy, Ravi Nair Physiotherapy College, Datta Meghe Institute of Higher Education and Research, Wardha, IND

**Keywords:** hemiparesis, innovative rehabilitation, neurological deficits, physical medicine and rehabilitation (pm&r), primary central nervous system lymphoma (pcnsl)

## Abstract

This case study examines the rehabilitation strategy for a 51-year-old farmer with primary neoplasm of the central nervous system (CNS)-related hemiparesis, balance issues, and cognitive impairment. Primary neoplasm of the CNS is a rare type of cancer that affects the brain, spinal cord, and other parts of the CNS. Hemiparesis, which is weakness on one side of the body, is a common symptom of primary neoplasm of the CNS. The tumour can cause inflammation and swelling in the brain, which can further contribute to weakness. Symptoms include headaches, confusion, seizures, and changes in vision or speech. The patient underwent surgical excision, chemotherapy, and radiation therapy but faced challenges in physiotherapy. The patient's initial assessment revealed asymmetries and impairments on the right side, including muscle weakness, flexor synergy, trunk imbalance, gait abnormalities, and cognitive impairment. A tailored physiotherapy protocol was implemented, focusing on improving muscle strength, synergy patterns, balance, gait, motor control, speech, and cognitive function. Innovative robotic gloves technology was incorporated to enhance hand functionality. This case study contributes to the growing body of evidence supporting the effectiveness of comprehensive rehabilitation strategies, including innovative technologies, in optimising recovery for individuals with CNS lymphoma-related neurological deficits. Further research and exploration could further validate their benefits and enhance the overall rehabilitation journey for such patients.

## Introduction

Primary neoplasm of the central nervous system (CNS) is a rare type of cancer that affects the brain, spinal cord, and other parts of the CNS [1,2]. Hemiparesis, which is weakness on one side of the body, is a common symptom of the primary neoplasm of the central nervous system [3]. The tumour can cause inflammation and swelling in the brain, which can further contribute to weakness [3]. Hemiparesis can occur due to the tumour compressing or invading the motor cortex, which is responsible for controlling voluntary movements of the body [3]. Primary neoplasm of the CNS can occur in people of all ages, but it is more common in older adults, and specific to gender, it is more common in males than females [4]. The exact cause of the primary neoplasm of the CNS is not known, but it is believed to be related to a weakened immune system [5]. People with a history of immune system disorders, such as HIV/AIDS, are at a higher risk of developing primary neoplasms of the CNS [1,6]. The clinical features of the primary neoplasm of the CNS can vary depending on the location and size of the tumour. In addition to hemiparesis, other common symptoms of primary neoplasm of the CNS include headache, confusion, seizures, and changes in vision or speech [3,7]. Patients may also experience nausea, vomiting, and fatigue. The diagnosis of primary neoplasms of the CNS typically involves a combination of imaging tests, such as magnetic resonance imaging (MRI) and computed tomography (CT) scans, and a biopsy of the tumour [8]. Treatment for primary neoplasms of the central nervous system typically involves chemotherapy, radiation therapy, or a combination of both. The choice of treatment depends on the location and size of the tumour, as well as the patient's overall health.

Primary neoplasm of the CNS is a rare type of non-Hodgkin neoplasm that can affect the brain, eyes, meninges, or spinal cord without evidence of systemic disease. It accounts for approximately 3% to 4% of all CNS tumours. The annual incidence of CNSL is around seven cases per 1,000,000 people in the United States, with a male predominance occurring mostly in the sixth decade of life [9,10]. Exoskeleton devices are larger than robotic gloves. Robotic gadgets have a glove-like construction that encloses the paretic hand. These gadgets have straightforward functioning and help lessen joint stress on the hands because of their glove-like design [11]. Positive attributes of robotic gloves often include fine control, ample feedback, close cognitive and physical interactions, portability, compactness, and lightweight design. Physiotherapy improves fine motor skills by allowing the hand to adapt to objects' shapes [12]. Rehabilitation tasks aim to improve cortical motor representation in the brain and functional hand improvements, enabling patients to perform daily activities independently. Robotic devices can enhance therapy quality [13]. Clinical research shows that patients using robotic assistance for physical hand therapy have better hand-motor skill recovery compared to those without it. The robotic device is used to improve distal motor function by enhancing active range of motion (AROM) and reducing spasticity around the wrist. The device is based on motor learning principles and provides feedback during repetitive activities to transfer functional distal motor activities. It offers consistent and precise therapy without fatigue, enhancing the quantity and quality of afferent information during consistent and larger amplitude movements [14,15].

## Case presentation

Patient information

A 51-year-old male farmer was admitted to the hospital on December 20, 2023, with complaints of weakness over the right upper limb and lower limb and difficulty in balancing for one year. The patient's son was the informant. The patient was evaluated on December 27, 2023. The patient also had secondary complaints of difficulty performing activities of daily living (ADLs) like bathing, toileting, and dressing.

The patient was apparently alright until May 2022. He started experiencing weakness on his right side. He had difficulty in calculation, after which the patient was taken to a nearby local hospital, where he was diagnosed with dementia and was prescribed some medications for dementia. After taking those medications, patients started getting vomiting episodes, and the patient's condition started getting worse. On June 4, 2022, the patient was not able to get up from his bed; then, immediately, he was taken to a private hospital in Nagpur. In Nagpur, some investigations, like MRI brain and CT brain, were done. An MRI showed a large, moderately enhancing soft tissue lesion in the left temporal and frontal lobe with the largest symmetrical white matter oedema and a significant mass effect compressing the left lateral ventricle and midline shift to the right (lesion: 4.8 ×4×6 cm). On June 6, 2022, a surgical excision was performed. After 15 days of surgery, the patient started taking chemotherapy and radiation therapy for three months. In September 2022, the patient was referred for physiotherapy sessions, which continued till February 2023. However, due to some circumstances, he could not continue the sessions. After nine months, i.e., on December 20, 2023, the patient came to AVBRH with a complaint of difficulty lifting the right upper and lower limbs and difficulty balancing, for which the patient was referred to the neuro-physiotherapy department on December 20, 2023.

Clinical findings

Upon observation, the patient was standing with several asymmetries and impairments, predominantly on the right side. The right shoulder was slightly elevated, accompanied by a slightly flexed elbow and pronated forearm. Additionally, the right wrist appeared flexed, with the fingers and thumb on the right side exhibiting flexion. Moreover, asymmetry in the right hip with slight internal rotation and a slightly flexed right knee were observed. Involuntary movement patterns revealed a flexor synergy pattern, while speech was characterized by slurring and frequent disruptions in fluency and flow. The gait pattern observed was identified as a circumductory gait. Neurologically, the patient was conscious but lacked orientation to time, place, or person and demonstrated cooperation during the examination. The Mini-Mental State Examination (MMSE) yielded a score of 13 out of 30, with notable deficits in orientation (2/5), attention and calculation (1/5), and copying (0). Cranial nerves were intact, and sensory examination revealed no abnormalities. Muscle tone grading using the modified Ashworth scale indicated a grade 2 increase in muscle tone through most ranges of motion for the right upper and lower limbs, while the left side showed no increase in muscle tone (grade 0). Balance and coordination assessments revealed a grade 1 on non-equilibrium tasks and a grade 1 on equilibrium tasks (postural control and balance tests). Reflexes are mentioned in Table 1.

**Table 1:** Reflexes +++ - Brisk reflex ++- Normal reflex

Reflexes	Right	Left
Biceps Reflex	+++	++
Triceps Reflex	+++	++
Brachioradialis Reflex	+++	++
Patellar Reflex	+++	++
Achilles Reflex	+++	++

Investigations

**Figure 1:**
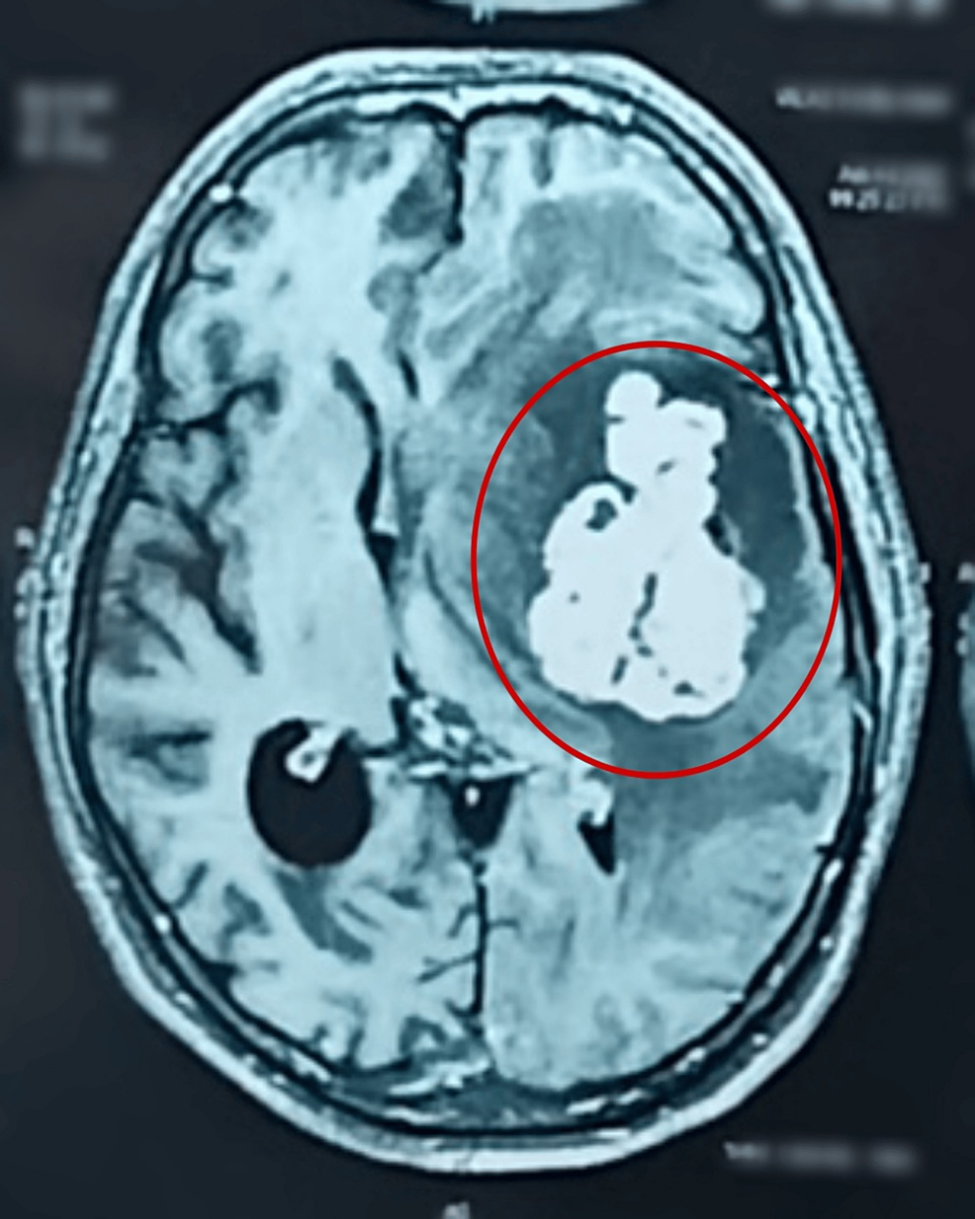
MRI of the brain

MRI revealed that there is a large moderately enhancing solid soft tissue lesion in the left temporal and frontal lobes with the largest symmetrical white matter oedema and significant mass effect compressing the left lateral ventricle and midline shift to right. Features are likely of a neoplastic lesion rather than a metastatic deposit. Since no evidence of metastasis was found on the body CT, it suggests that the neoplasm in the CNS is likely primary, originating within the brain rather than spreading from another location in the body. The impression is shown by a circle in Figure 1.

Physiotherapy rehabilitation protocol 

**Figure 2:**
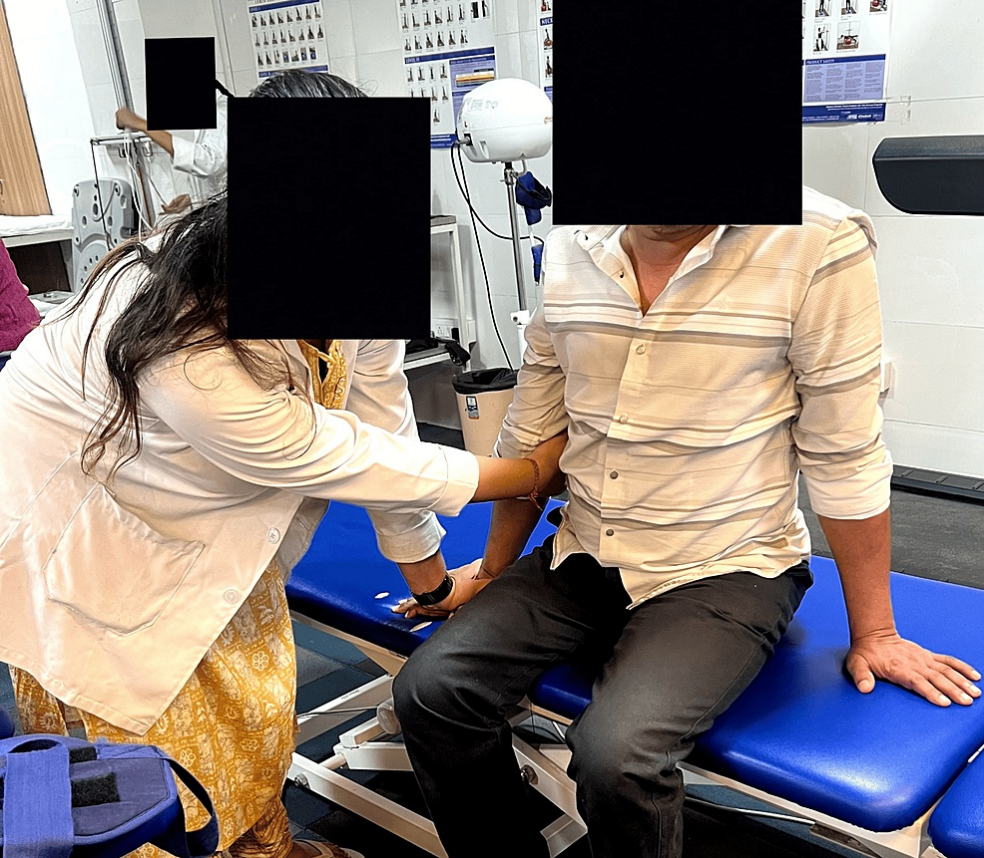
Weight shifting in sitting for balance

**Figure 3:**
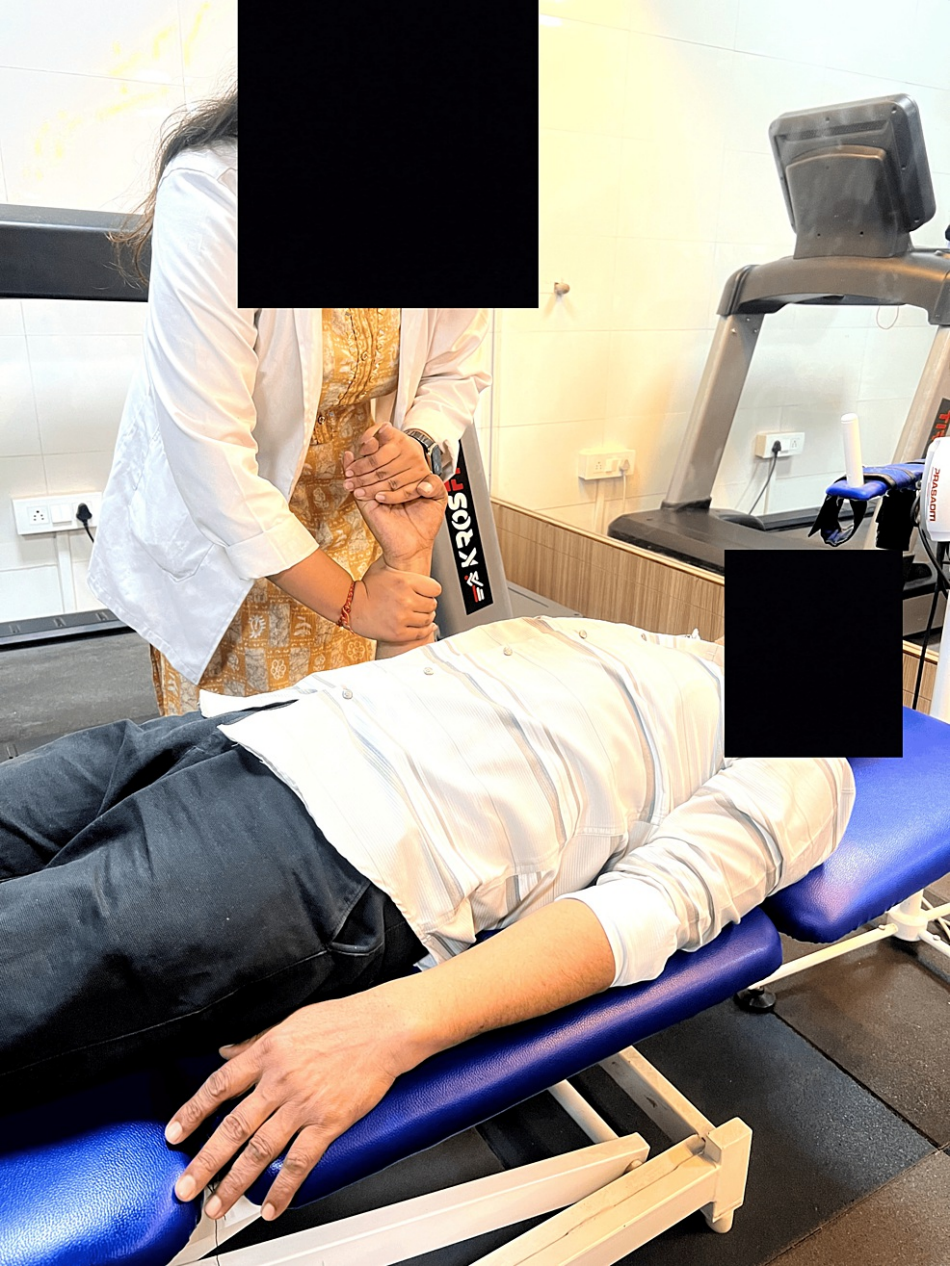
Proprioceptive neuromuscular facilitation

**Figure 4:**
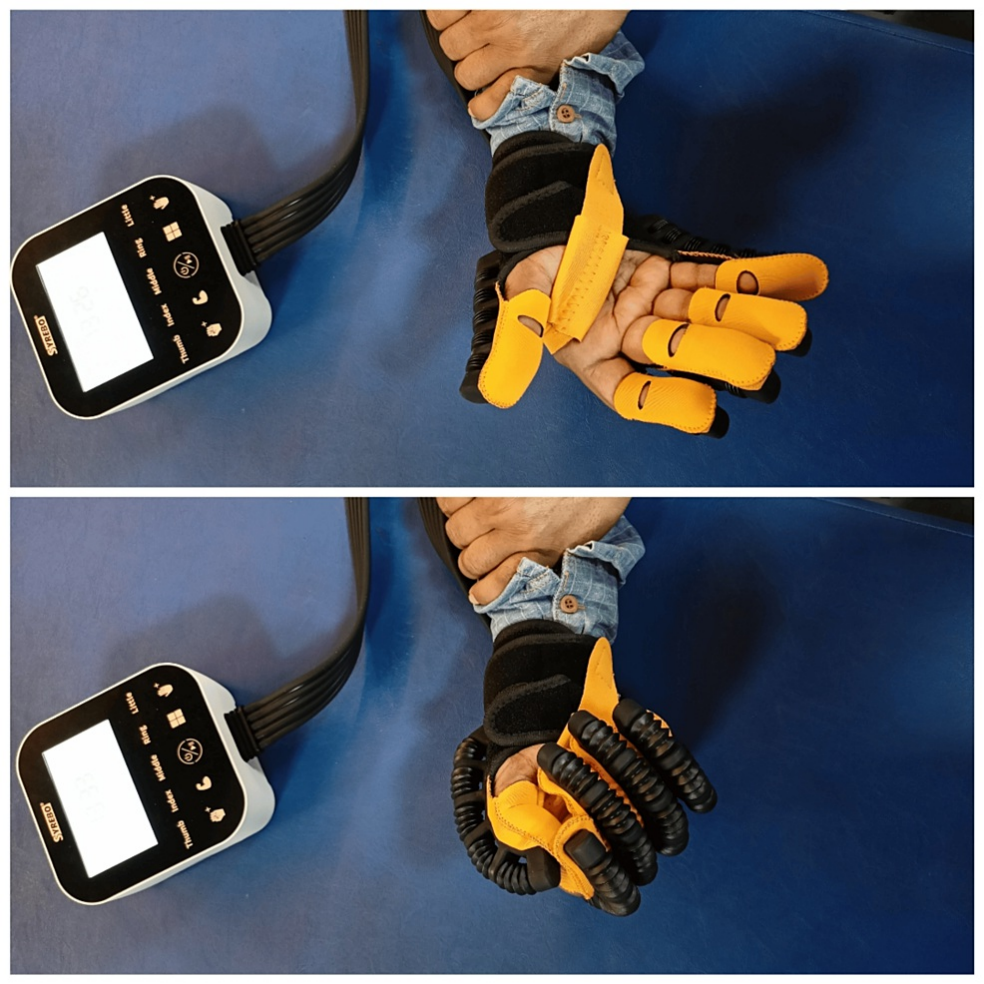
Robotic hand gloves rehabilitation

Table 2 shows the physiotherapy rehabilitation protocol. In the treatment, Figure 2 shows weight shifting in sitting for balance. Figure 3 shows proprioceptive neuromuscular facilitation, and Figure 4 shows rehabilitation using robotic hand gloves. 

**Table 2:** Physiotherapy rehabilitation protocol

Problem	Goals	Intervention	Dosage	Rationale
Muscle weakness	To improve strength in right limbs	Resistance exercises for Right lower limb and upper limb (this is done by using weight cuffs of ½ kg initially) 3 sets, 12 repetitions, proprioceptive training.	Four sessions/week	Enhance muscle control and functional abilities
Flexor synergy of upper limb	Enhance voluntary movement patterns	Neuromuscular re-education, Task-specific exercises (reach outs given in proprioceptive neuromuscular facilitation pattern and it should be actively assisted)- 10 reps ×3 sets	Daily exercises	Promote normal movement patterns
Trunk balance	To improve balance while sitting	Sitting Weight shifting, reach outs in multidirectional given in the sitting position.	Two sessions/day	Improve balance while sitting
Gait abnormalities	Improve walking ability and gait pattern	Gait training in a parallel bar, then on the trade mill for 10 minutes. Balance exercises (4-5 perturbation while standing with eyes closed)	Two sessions/day	Enhance walking coordination and stability
Motor control and coordination deficits	Improve coordination and functional movements	Coordination exercises (buttoning a shirt or writing) for gross motor skills include lifting activities and walking in straight line. Mirror therapy is also given	Four sessions/week	Enhance movement control and coordination
Speech difficulties	Enhance speech fluency and articulation	Speech therapy (vowels training), icing around tongue and mouth for speech improvement	Three sessions/week	Improve communication skills
Cognitive impairment	Enhance cognitive function	Cognitive exercises during therapy sessions, like card matching, put 3–4 pairs of cards placed on a deck facing downward and ask them to uncover two cards at a time and try to match pairs. This will help with mental recall and visual scanning.	Five sessions/week	Aid attention, memory, and problem-solving
Muscle tone abnormalities	Reduce increased muscle tone in right limbs	Stretching 20 seconds, hold each stretch for 3 sets, range of motion exercises for right upper limb and lower limb and PNF given in D1-D2 pattern	Two sessions/day	Enhance flexibility and movement
Flexor synergy of wrist and fingers	Improve hand functionality and reduce flexor synergy in the wrist and fingers.	session lasting around 15 minutes to 20 minutes, the patient will wear the robotic gloves and a timer set for 15 minutes.	2 to 5 times per week	To enhance the patient's ability to perform daily activities and regain independence.

Outcome measures

After giving 12 weeks of intervention, there were positive outcomes seen, which showed improvement. Table 3 shows outcome measures.

**Table 3:** Outcome measures 1: Slight increase in muscle tone manifested by a catch and release or by minimal resistance at the end of the range of motion when the affected part is moved in flexion or extension. 2: More marked increase in muscle tone through most of the ROM, but the affected part easily moved

Outcome Measure	Pre-treatment Score	Post-treatment Score
Berg Balance Scale	36/56	46/56
Functional Independence Measure (FIM)	93/108	104/126
Modified Ashworth Scale (for Right upper and lower limbs)	2	1

## Discussion

The post-treatment outcomes, showcasing considerable improvement in muscle tone and functional scores, in response to the comprehensive physiotherapy regimen and VR therapy, present an encouraging perspective on the rehabilitative potential for patients with CNS lymphoma-related deficits. The reduction in muscle tone abnormalities, particularly in the right upper and lower limbs, from grade 2 to grade 1 on the Modified Ashworth Scale aligns with previous studies highlighting the efficacy of stretching, range of motion exercises, and proprioceptive neuromuscular facilitation in improving muscle tone in neurological conditions. Studies such as those by Wang et al. (2016) emphasize the impact of these interventions in reducing spasticity and enhancing flexibility in stroke survivors [16]. Miller et al. (1997), in their study, selected nine participants, ranging in age from 47 to 71, who were diagnosed with left hemiparesis and right-sided stroke. Participants were gathered from an adjacent rehabilitation facility and a nearby hospital. Evidence from this study suggests that resistive training may actually result in reduced co-contraction and better control over force production. Individuals with pre-morbid decreased peak force levels may benefit the most from this kind of training. Therefore, strength training is more beneficial for the weakness of the upper and lower limbs [17]. The improvements in balance, indicated by the increase in Berg Balance Scale Score from 36/56 to 46/56, resonate with findings demonstrating the efficacy of balance exercises, weight shifting manoeuvres, and gait training in enhancing postural control and stability in patients with neurological impairments. Research by Tsaklis et al. (2012) underlines the significance of balance training in ameliorating gait abnormalities and reducing fall risks in individuals with neurological conditions [18]. The Functional Independence Measure (FIM) score enhancement from 93/108 to 104/126 is indicative of the broader functional improvements achieved through the comprehensive rehabilitation program. Studies such as the one by Fong et al. (2001) emphasize the relevance of multifaceted interventions, including motor control exercises and cognitive training, in enhancing functional independence and daily living activities in neurological patients [19]. Kutner et al. in their study evaluated the change in patient-reported, health-related quality of life linked with robotic-assisted treatment paired with less therapist-supervised training. Thirty hours of robotically aided treatment and sixty hours of therapist-supervised repeated task practice were contrasted. When combined with intense task practice therapies, robotic-assisted therapy might be a useful addition to or replacement for improving hand function recovery in stroke patients [20].

These outcomes align with existing literature, indicating the efficacy of physiotherapy interventions and the integration of innovative technologies like robotic hand gloves in optimizing recovery and functional outcomes in patients with CNS-related neurological deficits. Continued research and clinical exploration in this realm could further validate and elucidate the benefits of such interventions in enhancing the rehabilitation journey of patients affected by CNS lymphoma-related impairments.

## Conclusions

This case study demonstrates the successful rehabilitation of a 51-year-old farmer with primary neoplasm of CNS-related hemiparesis, balance issues, and cognitive impairment. Despite facing challenges in traditional physiotherapy, a tailored protocol integrating conventional techniques and innovative technologies like robotic gloves led to significant improvements in muscle tone, balance, functional independence, and cognitive function. The use of robotic gloves was particularly effective in addressing upper limb weaknesses and flexor synergy. Future validation and clarification of the advantages of using innovative equipment in rehabilitation programs might be achieved by future study and research in this area. The case study demonstrates the efficacy of these all-encompassing techniques and highlights how they can improve the entire rehabilitation process for patients who are dealing with impairments related to primary neoplasm of the CNS.
